# Psychosocial determinants of fruit and vegetable intake in adult population: a systematic review

**DOI:** 10.1186/1479-5868-7-12

**Published:** 2010-02-02

**Authors:** Laurence Guillaumie, Gaston Godin, Lydi-Anne Vézina-Im

**Affiliations:** 1 Research Group on Behaviour and Health, Laval University, Quebec City, Canada; 2 Laboratoire de psychologie EA 3188, Université de Franche-Comté, France; 3 Canada Research Chair on Behaviour and Health, Laval University, Quebec City, Canada

## Abstract

**Background:**

Accumulating evidence suggests that fruit and vegetable intake (FVI) plays a protective role against major diseases. Despite this protective role and the obesity pandemic context, populations in Western countries usually eat far less than five servings of fruits and vegetables per day. In order to increase the efficiency of interventions, they should be tailored to the most important determinants or mediators of FVI. The objective was to systematically review social cognitive theory-based studies of FVI and to identify its main psychosocial determinants.

**Methods:**

Published papers were systematically sought using Current Contents (2007-2009) and Medline, Embase, PsycINFO, Proquest and Thesis, as well as Cinhal (1980-2009). Additional studies were identified by a manual search in the bibliographies. Search terms included fruit, vegetable, behaviour, intention, as well as names of specific theories. Only studies predicting FVI or intention to eat fruits and vegetables in the general population and using a social cognitive theory were included. Independent extraction of information was carried out by two persons using predefined data fields, including study quality criteria.

**Results:**

A total of 23 studies were identified and included, 15 studying only the determinants of FVI, seven studying the determinants of FVI and intention and one studying only the determinants of intention. All pooled analyses were based on random-effects models. The random-effect R^2 ^observed for the prediction of FVI was 0.23 and it was 0.34 for the prediction of intention. Multicomponent theoretical frameworks and the theory of planned behaviour (TPB) were most often used. A number of methodological moderators influenced the efficacy of prediction of FVI. The most consistent variables predicting behaviour were habit, motivation and goals, beliefs about capabilities, knowledge and taste; those explaining intention were beliefs about capabilities, beliefs about consequences and perceived social influences.

**Conclusions:**

Our results suggest that the TPB and social cognitive theory (SCT) are the preferable social cognitive theories to predict behaviour and TPB to explain intention. Efficacy of prediction was nonetheless negatively affected by methodological factors such as the study design and the quality of psychosocial and behavioural measures.

## Background

Accumulating evidence suggests that fruit and vegetable intake (FVI) plays a protective role against major diseases. First, FVI decreases cardiovascular disease risk and especially cerebrovascular accident, given its content in micronutrients, antioxidant and phytochemical compounds and fibers [1-7]. Second, FVI decreases the risk of certain cancers, mainly of the digestive system [8,9]. Subjects with a low FVI are 1.5 to 2 times more at risk to develop a cancer than subjects with a high FVI [8-10]. Third, FVI is inversely linked to body weight [11-15] and fat mass [16-18]. Because of its low energy density in comparison with high sugar and high fat foods, FVI contributes to the prevention of weight gain among overweight individuals [19-25]. Finally, high FVI is associated with better health [26], especially among those who consume at least five servings per day [27]. More recently, some countries such as Canada have considered increasing the recommended FVI to at least seven servings per day for women and eight for men [28]. Australia also launched the Go for 2&5 campaign to promote a daily intake of two servings of fruits and five servings of vegetables [29].

Despite the protective role of FVI and the obesity pandemic context, populations in Western countries and all over the world usually eat far less than five servings per day [30,31]. It is thus important to develop effective interventions to promote their intake. In order to increase the potential efficiency of such interventions, they should be tailored to the most important determinants or mediators of FVI [32-34]. Two systematic reviews have analyzed the determinants of FVI in adult populations, but did not draw clear conclusions on this issue [32,35]. Baranowski et al. [32] reviewed qualitative and quantitative studies pertaining to the correlates and determinants of FVI. They observed several contradictory findings in the correlates of FVI, making it difficult to suggest clear recommendations about the psychosocial models that should be used and the mediating variables that interventions should target. These authors also concluded that most psychosocial models predict generally less than 30% of the variance in FVI which they qualified as "low predictiveness"; R^2 ^was higher for the models predicting narrow-food categories (e.g. milk or salad consumption). A decade later, Shaikh et al [35] led another review of scientific literature on psychosocial predictors of FVI. They analyzed the results of 35 studies (14 longitudinal and 21°cross-sectional studies) published between 1994 and 2004 that described the relationship between psychosocial predictors and adult FVI, and qualitatively rated their strength of evidence. They found strong evidence for self-efficacy, social support and knowledge as predictors of FVI. In addition, they found weaker evidence for perceived barriers, intention and attitudes to predict FVI among adults. However, in that review, broad definitions of few criteria overshadowed important aspects. First, "adult population" was a broad concept that included college students as well as elders. Second, in their analysis, the authors considered that very similar psychosocial constructs of different psychosocial models were different. In fact, there is a substantial overlap among constructs from different psychosocial models (e.g. outcome expectancies in the social cognitive theory (SCT), benefits in the health belief model (HBM), pros and cons in the transtheoretical model (TTM), attitude in the theory of planned behaviour (TPB))" [36]. Third, heterogeneity in designs and statistics in the included studies precluded the calculation of meaningful effect sizes and made it difficult to draw clear conclusions. Finally, they included longitudinal studies on determinants of FVI following a psychosocial intervention as well as studies on determinants of FVI without a psychosocial intervention. This latter point is important because any intervention can modify the relationships between determinants and the studied behaviour.

It was thus the aim of this study to review the determinants of FVI as well as the determinants of intention to eat fruits and vegetables. Cross-sectional and longitudinal studies based on social cognitive theories pertaining to the general adult population (excluding students and elders) and reporting the needed statistics for numerical calculation of effect size (R^2^) were included. The statistical results obtained were reviewed in relation with the variables tested and the theoretical models used.

More specifically, this review analyzed the scientific literature in order to provide information on a number of issues such as what is the overall average explained variance in FVI and intention, what psychosocial constructs predict FVI and intention, and what are the methodological moderators of the efficacy in prediction? Answering these questions is important considering that psychosocial interventions aimed at improving health-related behaviours should be tailored to the most important determinants or mediators of these behaviours.

## Methods

### Inclusion and Exclusion Criteria

We included studies that assessed the predictive value of social cognitive theories (e.g. theory of planned behaviour, social cognitive theory, etc.) using the R^2 ^statistic for FVI in the general adult population. Predictive studies included both longitudinal and cross-sectional studies focusing on prediction of behaviour. In the longitudinal studies behaviour is predicted at a later point in time, following the assessment of the potential psychosocial predictors. In cross-sectional studies, the measures of behaviour and psychosocial predictors are taken at the same time. These latter studies were included although longitudinal studies are considered more respectful of theoretical assumptions of the majority of the social cognitive theories [32,37]. Studies predicting FVI or intention following a health promotion intervention were not included. Also, studies among elders (>65 years of age), children (<18 years of age), students or seriously ill population were excluded. Finally, one longitudinal study measuring behaviour eight years after the measurement of psychosocial variables was considered an outlier and therefore excluded.

### Literature Search

Studies were identified by searching through electronic databases. This search was applied to Current Contents (2007-2009) and Medline, Embase, PsycINFO, Proquest dissertations and Thesis, as well as Cinhal (1980-2009). The last search was run on July 28, 2009. A combination of MeSH-terms and keywords was used: (Fruit OR Vegetable) AND (Behaviour OR Intention OR Psychosocial theory OR Planned behaviour OR Social cognitive theory OR Bandura OR Triandis). The leading author undertook the initial screen of the search results for potentially included studies. In addition, previous literature reviews and reference lists of included studies were manually checked.

### Review Methods

Two of the authors independently extracted data using a data extraction sheet. Disagreements were resolved by discussion and if no agreement could be reached, the third author made the final decision. Information was extracted from each included study on authors and year of publication, population under study, sample size, study design (longitudinal or cross-sectional), social cognitive theory used, behaviour under study (fruit intake: FI, vegetable intake: VI, or FVI), variables predicted (intention or behaviour), psychosocial variables assessed, main results and criteria of methodological quality.

Main results included the R^2 ^statistic, the variables entered in regression models and the variables which contributed significantly (p < 0.05) to the prediction of the dependant variable. The weight and p values of the significant variables were also documented. The variables were classified according to the theoretical domains defined by Michie and colleagues with the addition of taste and health value as additional categories [38] (Additional file 1 presents the classification of variables). This classification was evaluated as particularly useful recognizing there is a substantial overlapping among constructs from different psychosocial models [32,39,40].

Methodological quality was assessed using four criteria. For psychometric qualities of behavioural measure (first quality criteria), we dichotomized a validated tool as good versus poor if not validated or no information provided. For psychometric qualities of psychosocial measures (second quality criteria), we dichotomized the internal consistency values reported as good (Cronbach's alpha coefficient of 0.60 or more) versus poor if lower than 0.60 values or no information was reported [41]. If only partial information was provided, the studies were qualified as good if the reported psychometric qualities met the standards. The level of correspondence between intention and behaviour (third quality criteria) was evaluated according to Fishbein and Ajzen's guidelines [37]; intention and behaviour must correspond in terms of action (e.g. to eat at least 2 portions), target (e.g. fruit), context (usually not defined) and time (e.g. every day in the next week). Studies for which the measurement of intention and behaviour corresponded in terms of action and target were classified as having a good intention-behaviour level of correspondence. In case intention was not measured in a given study, the level of correspondence was considered good when most of the direct psychosocial constructs and behaviour corresponded in terms of action and target. The time and context elements were not considered. The study design (longitudinal versus cross-sectional) was also documented (fourth quality criteria).

### Methods of Analysis

Few studies used the same sample to predict different behaviours or to predict the same behaviour in different sub-samples. For instance, if several behaviours (e.g. raw vegetables, boiled vegetables) in a same category (e.g. vegetable intake) were predicted using a same sample, we randomly selected one of them. Also, if the prediction of a behaviour was available in different sub-samples (e.g. women, men), we randomly selected one of them. These decisions were taken before analyzing the data set in order to avoid attributing more weight to such studies.

For the analysis, we calculated an adjusted R^2 ^for each study correcting for the sample size and the number of predictors entered in the final regression model. This was done in order to avoid inflating the relative performance of some models over more parsimonious ones. Then, based on adjusted R^2^, a random-effect R^2 ^was calculated for the prediction of behaviour and intention in relation with FVI, FI only and VI only. The fixed-effects model assumes that the observed differences in results across studies reflect random variations and is used when there is a common effect for all included studies [42]. The random-effects model assumes that there is no common effect for all included studies; the variation of the effects across studies rather follows a particular distribution. In the presence of demonstrated between-study heterogeneity, we used the random-effects model as the main assumption of fixed-effects model was violated [42]. We assessed between-study heterogeneity using two common statistical approaches: a chi-squared test (Cochran's Q) and the I^2 ^statistic representing the percentage of total variation in estimated effects across studies that is due to heterogeneity rather than chance [42]. Sensitivity analyses were pre-specified. We compared the impact of a number of a priori defined potential moderators by comparing random-effect R^2 ^for different categories of moderators using Fisher's Z transformation procedures for correlations. Moderators included the main theory used (e.g. theory of planned behaviour, social cognitive theory, etc.), the sex of participants, and the four methodological quality criteria (psychometric quality of psychosocial and behavioural measures, level of correspondence between intention and behaviour and study design).

We also documented the variables measured to predict the dependant variables and the number of time these variables contributed significantly (p < 0.05) to the prediction of the dependant variables. Based on these data, a ratio was calculated (ratio = number of time significant/number of time assessed × 100). If there were missing data on the variables significantly predicting the dependant variable, the concerned study was excluded from the ratio analysis.

## Results

### Characteristics of the Included Studies

**Figure 1:**
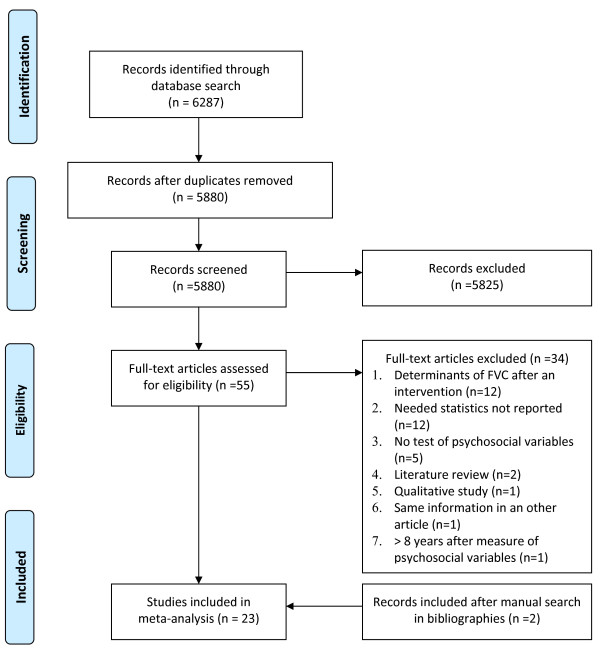
**Flow Diagram**.

Results from the bibliographic screening are presented in Figure 1. A total of 23 studies were included, involving 34,577 participants. All studies selected for the review were published in English between 1995 and 2008 and carried out in United States [43-57], Netherlands [58-61] and Great-Britain [62-65] (see Additional file 2). The majority of studies were based on samples of men and women (14 studies) [43,45,49,50,54,56,57,59-65]. The others were conducted among women only [44,48,52,53,58] or men only [46,47,51,55]. Twenty-two studies focused on determinants of behaviour (FI, VI or FVI) and seven on determinants of intention (FI, VI or FVI) [50,58-60,62,63,65]. Among studies focusing on determinants of behaviour, 12 studies used a multicomponent theoretical framework, i.e. measuring variables from several social cognitive theories (e.g. social cognitive theory, transtheoretical model, health belief model, social support theory, theory of reasoned action) [46-55,57,64], seven studies the Ajzen's theory of planned behaviour (TPB) or the related attitude-social influence-efficacy (ASE) [66] model [58-63], three used Bandura's social cognitive theory (SCT) [43,44,56] and one was based on the health belief model (HBM) [45]. Among the studies which focused on determinants of intention (7 studies), six used the TPB framework [58-60,62,63,65] and one used a multicomponent framework [50]. Three quarter of the studies (18 studies) adopted a cross-sectional design to study the determinants of behaviour and only five studies used a longitudinal design [43,59,61-63]. In these latter studies, the time interval between the baseline measurement of psychosocial variables and behaviour assessment ranged between one and five weeks. There were 18 studies using good quality instruments to measure psychosocial variables but five studies were evaluated as presenting poor quality or no information was given in the manuscript [46,49,52,54,65]. Of the 22 studies which measured behaviour, two used a poor quality behavioural measure instrument or no information was provided [45,51]. Finally, of the 16 studies which studied determinants of FVI, one third demonstrated a low level of correspondence between predictors and behaviour [43,46,48,54,62]. However, among studies of the determinants of FI only or VI only, most of them (7 studies) demonstrated a low level of correspondence between predictors and behaviour [44,47,49,51,52,54,57]. Among the 16 studies which focused on determinants of FVI, half included between three and nine variables in their regression models [43,45,46,50,55,62-64]. In the other half (8 studies) between 13 and 32 variables were included in the models tested [44,48,49,51,53,54,56,57]. Similar proportions were observed for studies on determinants of FI only and VI only. A summary of the studies is presented in additional files. Additional file 3 summarizes studies aimed at predicting FVI whereas Additional files 4 and 5 respectively summarize FI and VI. Additional file 6 summarizes studies explaining intention to eat fruits and vegetables.

### Synthesis of Results

There were important variations in efficacy of behaviour prediction; the adjusted R^2 ^varied between 0.06 and 0.61 for FVI, 0.07 and 0.39 for FI and 0.07 and 0.32 for VI (see Additional files 3, 4 and 5, respectively). Similar variations in explained variance in intention were observed; it varied between 0.14 and 0.68 for FVI, 0.35 and 0.49 for FI and 0.13 and 0.51 for VI (see Additional file 6). In the pooled analysis, the random-effect R^2 ^for the prediction of behaviour was respectively 0.23, 0.19 and 0.14 for FVI, FI and VI. Regarding the prediction of intention, the random-effect R^2 ^was respectively 0.34, 0.43 and 0.31 for FVI, FI and VI.

The overall efficacy of prediction according to the main theory used to guide the studies is presented in Table 1. For the prediction of FVI, the most often used theoretical frameworks were a multicomponent framework, SCT and TPB. In the pooled analysis, the random-effect R^2 ^for the prediction of FVI using multicomponent framework was 0.16 (10 studies, n = 28,090) and 0.37 for the prediction of FVI using other models (SCT, TPB, HBM) (6 studies, n = 3,785). The predictive power of studies employing only one theory to predict FVI was significantly better than studies employing a multicomponent framework (Z = 16.73; p < 0.001). The random-effect R^2 ^for the prediction of FVI using SCT was 0.41 (3 studies, n = 2,286) and 0.45 for studies based on the TPB (2 studies, n = 430). However, the predictive power of studies employing the SCT or the TPB to predict FVI was significantly better than studies not employing the SCT (Z = 12.90; p < 0.001) or the TPB (Z = 6.40; p < 0.001). The predictive power of studies based on the TPB or SCT did not differ (Z = 0.95; p > 0.05). Finally, it was verified if using a given theory (e.g. HBM) alone or in a multicomponent framework affected the efficacy of prediction; similar results were obtained.

**Table 1:** Overall Efficacy of Prediction According to the Theoretical Framework Used

	Fruit and Vegetable Intake		Fruit Intake		Vegetable Intake	
	
	Nb of Participants (Nb of Studies)	Random-Effect R^2^	Nb of Participants (Nb of Studies)	Random-Effect R^2^	Nb of Participants (Nb of Studies)	Random-Effect R^2^
**Overall Efficacy of Prediction**						
...to model behaviour	31875 (16)	0.23	9120 (11)	0.19	8337 (9)	0.14
...to model intention	16717 (3)	0.34	1894 (4)	0.43	1267 (3)	0.31
**Efficacy of Frameworks to Model Behaviour**						
SCT	2286 (3)	0.41	794 (1)	0.17	794 (1)	0.13
TPB	430 (2)	0.45	1309 (4)	0.28	526 (2)	0.10^1^
HBM	1069 (1)	0.16	-	-	-	-
Multicomponent	28090 (10)	0.16	7017 (6)	0.15	7017 (6)	0.15
**Efficacy of Frameworks to Model Intention**						
TPB	430 (2)	0.41	1894 (4)	0.43	1267 (3)	0.31
Multicomponent	16287 (1)	0.21	-	-	-	-

Note: Dashes indicate that no study was concerned.

^1^Non-significant heterogeneity. Significant heterogeneity was detected using Q test (p < 0.05) and degree of heterogeneity I^2 ^> 75% in all other analyses.

For the prediction of FVI intention, only the TPB (2 studies, n = 430) and a multicomponent framework (1 study, n = 16,287) were used to model intention (see Table 1). The predictive power of the studies employing the TPB to explain FVI intention was significantly better than the study based on a multicomponent framework (Z = 5.44; p < 0.001). For the FI and VI intentions, only the TPB had been used (respectively for 4 studies, n = 1,894 and 3 studies, n = 1,267). The random-effect R^2 ^for the prediction of FI intention and VI intention was respectively 0.43 and 0.31.

### Moderators of the Efficacy of Prediction

The efficacy in prediction of behaviour and intention according to different methodological moderators is presented in Table 2. The results indicate that the prediction of behaviour (FVI and FI) was significantly better in studies using a longitudinal compared to a cross-sectional design (for FVI, Z = 14.56; p < 0.001; for FI, Z = 4.43; p < 0.001). No longitudinal study was carried out on VI. Concerning the level of correspondence in FVI prediction, for the studies where the level was high (11 studies, n = 27,057), a lower random-effect R^2 ^was observed (Z = 11.08; p < 0.001) compared to studies with a low level of correspondence (5 studies, n = 4818). An opposite result was obtained for FI: a higher random-effect R^2 ^was observed in studies having a high level of correspondence (Z = 6.15; p < 0.001). For VI, no difference was observed between studies with a low or a high correspondence level (Z = 1.82; p > 0.05). Concerning the psychometric quality of the instruments used to assess psychosocial variables, better predictions of FVI and FI were observed in studies with good reliability compared to poor reliability levels (Z = 8.5; p < .0001; Z = 6.83; p < 0.001, respectively); there were no significant differences for VI (Z = 0.18; p > 0.05). Concerning the quality of the behavioural measure, a better prediction of FVI and VI were observed in studies with good compared to poor psychometric qualities (Z = 5.7; p < 0.001; Z = 2.01; p < 0.05, respectively); no significant difference was observed for FI (Z = 1.5; p > 0.05).

**Table 2:** Overall Efficacy of Prediction of Behaviour According to Methodological Factors

	Fruit and Vegetable Intake		Fruit Intake		Vegetable Intake	
	
	Nb of Participants (Nb of Studies)	Random-Effect R^2^	Nb of Participants (Nb of Studies)	Random-Effect R^2^	Nb of Participants (Nb of Studies)	Random-Effect R^2^
**Study Design**						
Cross-sectional	30733 (13)	0.18	8337(9)	0.17	8337 (9)	0.14
Longitudinal	1142 (3)	0.51	783(2)	0.29	-	-
**Level of Correspondence**						
Low	4818 (5)	0.33	7811 (7)	0.15	7811 (7)	0.15
Good	27057 (11)	0.20	1309 (4)	0.28	526 (2)	0.10^1^
**Psychometric Quality of Psychosocial Variables**						
Low	6233 (3)	0.17	3680 (3)	0.12	3680 (3)	0.14
Good	25642 (13)	0.25	5440 (8)	0.22	4657 (6)	0.14
**Quality of Behavioural Measures**						
Low	1360 (2)	0.16^1^	291 (1)	0.18	291 (1)	0.08
Good	30515 (14)	0.25	8829(10)	0.19	8046 (8)	0.15
**Samples**						
Women and Men	25623 (10)	0.29	7224 (6)	0.22	6441 (4)	0.17
Women	2707 (3)	0.17	1164 (3)	0.20	1164 (3)	0.12
Men	3545 (3)	0.12	732 (2)	0.12	732 (2)	0.10

Note: Dashes indicate that no study was concerned.

^1^Non-significant heterogeneity. Significant heterogeneity was detected using Q test (p < 0.05) and degree of heterogeneity I^2 ^> 75% in all other analyses.

The efficacy of the studies to predict FVI according to gender of the samples was also analyzed (see Table 2). The comparison of the random-effect R^2 ^for FVI indicated that the prediction for samples combining women and men was better than samples based either exclusively on women (Z = 8.16; p < 0.001) or men (Z = 13.34; p < 0.001). The same significant contrasts were observed for VI. The prediction for samples combining women and men was better (compared to women Z = 2.35; p < 0.02; compared to men Z = 2.89; p < 0.01). The prediction of FI for samples combining women and men was better than for the samples of men only (Z = 3.8; p < 0.001).

### Most Consistent Variables Associated with Behaviour and Intention

The number of times a given variable was assessed in regression models and found to have a significant effect for the prediction of behaviour and intention is presented in Table 3. Among the variables assessed, the variables most consistently associated with the prediction of FVI (at least 50% of time) were habit, motivation and goals, beliefs about capabilities and knowledge. The same variables were also most consistently associated for FI and VI. For VI, however, there was an additional association with taste. It can be noted that behavioural regulation was assessed only once and was found significant in the FI, VI and FVI predictions. Consequently, this result has to be interpreted with caution. With respect to the factors explaining intention regarding FVI, the most consistently significant cognitive variables (i.e., at least 50% of the time) were beliefs about capabilities, beliefs about consequences and social influences. The same variables were also most consistently associated for FI and VI intention.

**Table 3:** Variables Measured and Associated with Behaviour and Intention

Variables Measured	Fruit and Vegetable Intake			Fruit Intake			Vegetable Intake		
	**Number of times**		**Ratio**	**Number of times**		**Ratio**	**Number of times**		**Ratio**
	
**Prediction of Behaviour**	**Assessed**	**Significant**	**(%)**	**Assessed**	**Significant**	**(%)**	**Assessed**	**Significant**	**(%)**
	
Habit	3	3	100	3	3	100	2	2	100
Motivation and goals	5	4	80.0	6	3	50.0	4	2	50.0
Beliefs about capabilities	13	10	76.9	10	7	70.0	8	5	62.5
Knowledge	8	5	62.5	4	2	50.0	4	2	50.0
Beliefs about consequences	11	5	45.4	9	3	33.3	8	2	25.0
Social influences	8	3	37.5	8	1	12.5	7	0	0
Context and life experiences	7	2	28.6	3	1	33.3	3	1	33.3
Taste	4	1	25.0	4	1	25.0	4	2	50.0
Sociodemographic variables	10	2	20.0	6	2	33.3	5	1	20.0
Social role and identity	2	0	0	1	0	N/A	1	0	N/A
Health Value	1	0	N/A	1	0	N/A	1	0	N/A
Behavioural regulation	1	1	N/A	1	1	N/A	1	1	N/A
**Prediction of Intention**									
Beliefs about consequences	2	2	100	4	3	75.0	3	3	100
Beliefs about capabilities	2	2	100	4	4	100	3	3	100
Social influences	2	1	50.0	4	3	75.0	3	2	66.7
Context and life experiences	1	0	N/A	0	0	N/A	1	0	N/A
Habit	0	0	N/A	1	1	N/A	0	0	N/A
Sociodemographic variables	0	0	N/A	1	1	N/A	0	0	N/A

Note: Ratio = (Significant/Assessed) × 100; Significant: p < 0.05; N/A: not computed because it was assessed only once; 20 studies enabling calculation of a ratio were included.

## Discussion

The present systematic review examined the efficacy of studies based on social cognitive theories to explain intention and predict FVI, FI and VI. It also verified the effect of various factors that could affect the efficacy in prediction. A small number of studies met the inclusion criteria and, surprisingly, far more research was carried out on determinants of behaviour than on determinants of intention. Overall, the proportion of variance explained for FVI, FI and VI was respectively 23%, 19% and 14%. These values are lower than the values reported in several meta-analyses of the TPB, the most widely used social cognitive model of health behaviour (27% in Armitage and Conner [67] and 34% in Godin and Kok [68]). Regarding the prediction of intention, the explained variance for FVI, FI and VI was respectively 34%, 43% and 31%. These are equivalent to those previously reported for applications of the TPB (39% in Armitage and Conner [67] and 40% in Godin and Kok [68]).

In scientific literature, it is suggested that theoretically informed programs are more effective in changing health behaviour than those that are not theoretically informed [39]. In the present study, the most frequently used theoretical frameworks were TPB, SCT, HBM and a multicomponent approach combining several theories. The theories used in the included studies are among the most widely used in the past two decades [69,70]. Our review suggests that the TPB and SCT perform well for the study of the determinants of FVI and that the TPB seems an appropriate choice to predict intention. The results also showed that the TPB and SCT were equivalent for behavioural prediction, probably due to the similarities in their constructs [40,71,72]. The HBM appears less appropriate to study FVI, most likely because illness avoidance and perceived threat are not salient issues for these behaviours or the population involved in the included studies [39,73]. Interestingly, our results showed that using a multicomponent theoretical framework performed significantly less well than using constructs from only one theory. One of the possible reasons to explain this result could be the failure to appropriately translate these multicomponent theoretical constructs into practice. As such these studies using multicomponent theoretical constructs are most likely "theory-inspired" instead of "theory-based". This phenomenon was previously identified by Michie et al. [74]. Also, derivations from the initial theory may misrepresent the theory or omit key components of it [40]. This is an interesting result in a context where researchers call "to empirically integrate salient components of different theories in an effort to create a more complete theory of behaviour change" [[75], p.292] and assume that "dietary behaviour is extremely complex and is, therefore, unlikely to be determined by one all-encompassing model" [[76], p.13]. Our results suggest that a multicomponent framework should ensure integration of salient determinants. To improve the prediction of behaviour and intention, it seems necessary to first rigorously apply theories that proved their effectiveness in the past and add promising constructs recently identified as contributing to the predictive models [40]. On this regard, innovating constructs pertaining to affective attitude (e.g. taste, satisfaction, pleasure), behavioural regulation (e.g. action control, action planning, action coping, self-regulation) or self-identity were rarely or not at all tested in the reviewed studies [39,77-80]. Such constructs could favourably be tested in future studies of the determinants of FVI.

The analysis of methodological factors indicated that the majority of the studies included were relying only on cross-sectional data. It must be acknowledged that it is not an appropriate method to identify causal determinants of behaviour [39,69,75,81]. Nonetheless, such studies provide some evidence whether psychosocial constructs are associated or not with intention or behaviour. However, higher efficacy in prediction when using a longitudinal design was observed than for a cross-sectional design. This is surprising given that cross-sectional designs are known to inflate the relationship between psychosocial variables and behaviour. This may partly be explained by the fact that studies using a longitudinal design may have given more considerations to methods. We obtained moderate support for the higher efficacy in prediction when good psychometric quality instruments were used to assess psychosocial and behavioural measures.

It was also noted that a higher proportion of variance in FVI was explained than in FI and VI (respectively 23%, 19% and 14%). This result contradicts the idea that a narrow definition of the studied behaviour is better explained than a broader behavioural category [32]. Considering the small number of studies included on FI and VI, it is difficult to explain these differences. However, it could be suggested that the methodological quality of studies played a significant role. For instance, the explained variance for FVI, FI and VI was inversely linked to the number of studies using a cross-sectional design (respectively 81%, 82% and 100%), to the number of studies presenting a low level of correspondence between intention and behaviour (respectively 31%, 64% and 78%) and to the number of studies presenting low psychometric qualities of the measurement of psychosocial variables (respectively 19%, 27% and 33%). We also noted that variability in FVI, FI and VI was better explained for samples combining men and women compared to samples of men or women only. It would be necessary to conduct additional studies to determine if these differences are due to methodological considerations or if other reasons could explain the lower prediction of FVI among the male samples. To our knowledge, no study or literature review has previously noticed a difference in the efficacy of prediction between genders. This should be given consideration in future studies.

Interventions are likely to be more effective if they target determinants of behaviour [82,83]. Thus, the first stage in developing interventions is to identify what predicts a given behaviour [40]. In this review, the most consistent variables associated with FVI, FI and VI were habit, motivation and goals, beliefs about capabilities and knowledge. Taste was also an important determinant of VI only. Consequently, these results provide support for the role of the main determinants of the TPB and SCT (motivation and goals and beliefs about capabilities) to predict behaviour [67]. Concerning the role of habit, several previous studies found that it was a good predictor of future behaviour [84,85]. Since FVI is performed daily and frequent performance can establish strong habits, these habits directly guide future behaviour [86]. The direct role of knowledge on behaviour is more surprising. Knowledge is usually predictive of a precautionary behaviour during the early stages of a health issue when many people are not yet aware of the threat. However, its influence declines as information becomes widespread [87,88]. Knowledge would consequently remain an important determinant to consider in future interventions. Finally, taste as a proximal measure of affective attitude has a direct effect on VI but not on FI. This finding suggests that affect can predict performance of specific behaviours and is a key to change VI [89]. More studies should investigate the role of affective constructs (e.g. taste, satisfaction, pleasure) in the prediction of FVI and eating behaviour in general. It was also noted that variables which significantly contributed to the prediction of eating behaviour in previous studies have not been often measured in studies of FVI. Social role and identity have been measured in only two studies, behavioural regulation only once and taste no more than four times. Anticipated regret or intention stability was not included in the reviewed studies. More studies applying theories are needed and also more innovative psychosocial measures are necessary to improve behavioural prediction.

**Figure 2:**
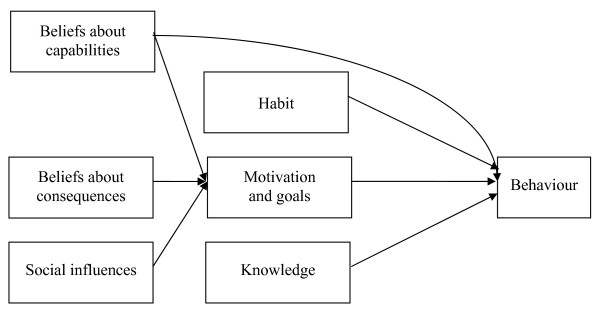
**Summary of the Relationships Observed between the Variables for the Theory-Based Studies Predicting Fruit and Vegetable Intake**.

The most consistent determinants of intention were beliefs about consequences, beliefs about capabilities and social influences. In agreement with previous reviews, although the importance of social influence on intention appears to be less important than beliefs about consequences and beliefs about capabilities, it remains in this review a significant determinant of intention [67,68]. The interrelationship of the variables in the prediction FVI and intention are summarized and illustrated in Figure 2. We do not imply that other factors are not important, but it appears from the present analysis that the integration of these variables presented in Figure 2 summarizes the majority of the observations. We noted that variables pertaining to context and life experiences or sociodemographic variables did not appear to be determinants of behaviour or intention. Moreover, no systematic analysis of mediators and moderators were carried out in the included studies.

### Limitations

One of the significant limitations of this systematic review is the small number of studies included. This limits the robustness of the current findings. This can be explained by the fact that only one third of the published studies on health behaviour used a theory [69] and that we did not include the grey literature. Moreover, publication bias (pertaining to small samples and interest in studies on determinants of behaviour) might also bias the sample and account for some of the effects that were observed.

Based on our results (number of predictors, explained variance), it can be suggested that some theoretical models seem to be more efficient to predict FVI or intention. Nonetheless, none of the included studies in this review compared the efficacy of different theories to predict FVI and, in general, very few studies have empirically compared different theories [40,77,90]. Moreover, in this review, it was assumed that a theory is successful when the explained variance is the highest. However, in using other criteria of success (clinical meaningfulness, intervention value, population or cultural specificity, parsimony, etc.), other conclusions could have been reached [75,82,91]. For instance, a theory might explain more variance than another but using variables that cannot be modified and therefore be less useful for interventions [81]. We also identified in this study the most consistent variables associated with behaviour or intention. However, the influence of psychosocial variables on intention or behaviour were based on significance ratio and relied on a null hypothesis testing and not on an estimate of the effect size [91].

Another limitation of this review is that it was not possible to ascertain whether the theories were used correctly [69,92]. Constructs may be misinterpreted or poorly measured and analyses may have been inappropriate. This may explain the poor performance of some studies [40].

## Conclusions

To our knowledge, this study is the first systematic review aimed at investigating psychosocial theories that should be used to study FVI and the mediating variables that interventions should target. Our review sought to provide information to researchers on which theories are the most fruitful to apply in predictive studies and to identify the variables explaining intention as well as FVI. Moreover, a number of methodological factors were identified as potential moderators of the efficacy in prediction. There is an urgent need for sound theoretically-based research on determinants of FVI. These future studies should rigorously apply the most effective psychosocial theories such as the TPB or SCT and test for promising but new variables in order to move the field forward. In particular, the role of affective attitude, behavioural regulation and social-identity should be investigated. Moreover, differences in efficacy of prediction according to gender and food category (FVI, FI or VI) should be explored. Future studies should also take into consideration methodological aspects such as study design in order to contribute to the development of a significant corpus of data on the FVI. This review also suggests that there is sufficient evidence on determinants of FVI to guide intervention development. Tailored interventions should target motivation and goals, beliefs about capabilities, knowledge, taste (especially for VI) and break the influence of habit. We hope that the information provided in this review of the scientific literature will be useful to researchers in the planning of studies that may lead to improved strategies to promote FVI.

## Competing interests

The authors declare that they have no competing interests.

## Authors' contributions

LG carried out literature search, data extraction, data analysis and drafted the manuscript. LAVI participated in data extraction. GG participated in discussing the paper, providing methodological input and helped to draft the manuscript. All authors read and approved the final manuscript.

## Supplementary Material

Additional file 1 **Classification of Variables**. This table describes the domains of the variables extracted for the review. Click here for file

Additional file 2 Descriptive Statistics of the Included Studies. Click here for file

Additional file 3 Summary of Studies Predicting Fruit and Vegetable Intake. Click here for file

Additional file 4 Summary of Studies Predicting Fruit Intake. Click here for file

Additional file 5 Summary of Studies Predicting Vegetable Intake. Click here for file

Additional file 6 Summary of Studies Explaining Fruit and Vegetable Intake Intentions. Click here for file
