# Supplementary material for: Psychosocial determinants of fruit and vegetable intake in adult population: a systematic review
Source: Int J Behav Nutr Phys Act. 2010 Feb 2;7:12. doi: 10.1186/1479-5868-7-12 (PMC2831029; doi:10.1186/1479-5868-7-12)
Supplement: Additional file 1 — Classification of Variables. This table describes the domains of the variables extracted for the review. [file 1479-5868-7-12-S1.DOC]

## Additional file 1 – Classification of Variables

| Domains | Constructs. |
| --- | --- |
| Knowledge | Knowledge regarding the recommended FVI and vitamins. Know how to select healthy foods. Nutrition awareness. Awareness of food guide pyramid. |
| Motivation and Goals | Intention. Stages of change. Readiness. |
| Beliefs about Consequences | Attitudes. Outcome expectations. Pros and cons. Perceived benefits. Perceived need. Perceived susceptibility to cancer. Nutrition concern. Predisposing factors. |
| Social Influences | Social support. Subjective norm. Social influences. Social norms. Encouragement. Interpersonal factors. |
| Beliefs about Capabilities | Perceived behavioural control. Self-efficacy. Barriers. Availability. Accessibility. Skills. Enabling factors. |
| Social Role and Identity | Modeling attitudes. Set example for others. |
| Health Value | Health as a value. |
| Behavioural Regulation | Self-regulation. Change strategies. |
| Taste | Like/dislike taste. Preferences. |
| Habit | Habit strength. Cooking habits. |
| Sociodemographic Characteristics | Age. Gender. Socio-economic status. Body mass index. Education. Income. Ethnicity. Family size. Employment status. Marital status. Number of children < 18. |
| Context and Life Experiences | Illness history. Self-rated health. Smoking status. Alcohol intake. Physical activity. Job control. Job demands. Site type. |
