# Supplementary material for: Psychosocial determinants of fruit and vegetable intake in adult population: a systematic review
Source: Int J Behav Nutr Phys Act. 2010 Feb 2;7:12. doi: 10.1186/1479-5868-7-12 (PMC2831029; doi:10.1186/1479-5868-7-12)
Supplement: Additional file 2 — Descriptive Statistics of the Included Studies. [file 1479-5868-7-12-S2.DOC]

## Additional file 2 – Descriptive Statistics of the Included Studies

| **Study Characteristics** | **Number of Participants** | **Number of Studies** | **% of Studies** |
| --- | --- | --- | --- |
| ***Among All Included Studies (23 studies, n = 34577)*** |  |  |  |
| **Country** |  |  |  |
| United States | 31826 | 15 | 65.2 |
| Great-Britain | 1442 | 4 | 17.4 |
| Netherlands | 1309 | 4 | 17.4 |
| **Date of Publication** |  |  |  |
| 2000-2008 | 8812 | 15 | 65.2 |
| 1995-1999 | 25765 | 8 | 34.8 |
| **Samples** |  |  |  |
| Women and men | 27514 | 14 | 60.9 |
| Women only | 3077 | 5 | 21.7 |
| Men only | 3986 | 4 | 17.4 |
| **Focus of Studies** |  |  |  |
| Determinants of behaviour and intention | 17870 | 6 | 26.1 |
| Determinants of behaviour only | 15966 | 16 | 69.6 |
| Determinants of intention only | 741 | 1 | 4.3 |
| ***Among Studies on Determinants of Behaviour***  ***(22 studies, n = 33836)*** |  |  |  |
| Pertaining to FVI | 31875 | 16 | 72.7 |
| Pertaining to FI | 9120 | 11 | 50.0 |
| Pertaining to VI | 8337 | 9 | 40.9 |
| ***Among Studies on Determinants of Intention***  ***(7 studies, n = 18611)*** |  |  |  |
| Pertaining to FVI | 16717 | 3 | 42.9 |
| Pertaining to FI | 1894 | 4 | 57.1 |
| Pertaining to VI | 1267 | 3 | 42.9 |
