# Supplementary material for: Psychosocial determinants of fruit and vegetable intake in adult population: a systematic review
Source: Int J Behav Nutr Phys Act. 2010 Feb 2;7:12. doi: 10.1186/1479-5868-7-12 (PMC2831029; doi:10.1186/1479-5868-7-12)
Supplement: Additional file 3 — Summary of Studies Predicting Fruit and Vegetable Intake. [file 1479-5868-7-12-S3.DOC]

## Additional file 3 – Summary of Studies Predicting Fruit and Vegetable Intake

| **Study** | **Population**  **(Sample Size)** | **Theoretical Framework** | **Measure of Behaviour** | **Behaviour Studied by psycho-social Variables** | **Design, Level of Corresp., Quality of BM, Quality of PSM** | **Variables Tested** | **R2**  **Significant Variables in the Final Model** |
| --- | --- | --- | --- | --- | --- | --- | --- |
| Anderson et al. 2007 [41] | US churchgoers  (n= 712) | SCT | FV servings per 1000 kcals (using Block-FFQ and food-shopping receipts) | To increase fiber and FV intake | Longit.  (10 to 14 days)  Low corresp.  Good BM  Good PSM | Beliefs about consequences (Positive outcome expectations, Negative outcome expectations), Social influences (Family social support), Beliefs about capabilities (Self-efficacy), Behavioural regulation (self-regulation), Sociodemographic characteristics (Socioeconomic status, Age, Gender) | **0.61**  Self-regulation (0.52 ***), Age (0.25***), Socioeconomic status (0.23***), Negative outcome expectations (-0.17**), Gender (-0.19***) |
| Cartwright 2003 [42] | US Low-income African American women  (n= 794) | SCT | Estimates of FV intake (7-item FV questionnaire | FV intake | Transver.  Good corresp.  Good BM  Good PSM | Knowledge, Beliefs about consequences (Health outcome expectancies), Beliefs about capabilities (FV availability at home, FV accessibility at home, FV accessibility away from home, Barriers, Preferences, Self-efficacy for eating 5 a day, Self-efficacy for eating FVJ when eating out, Self-efficacy for substituting high-fat food by FVJ, Self-efficacy for having FVJ available and accessible, Self-efficacy for shopping FVJ, Self-efficacy for cooking and preparing FVJ ), Social role and identity (Modeling attitudes), Habit (Low-fat cooking practices, High fat cooking practices, High-fat table practices), Health value (Health as a value), Taste (Juice preferences, Fruit preferences, Vegetable preferences), Sociodemographic characteristics (Age, Education, Employment status, Marital status, Family size), Context and life experiences ( Smoking status, Presence of current illness, Family member current illness, Family activity, Family communication, Parental involvement) | **0.21**  FV accessibility away from home (0.06*), Low-fat cooking practices (0.05*), FV availability at home (0.03**), , Self-efficacy (0.01**), High fat cooking practices (-0.05*) |
| Dittus et al. 1995 [43] | US adults  (n= 1069) | HBM | FV nutrition score (FFQ) | Intake of FV | Transver.  Good corresp.  Low BM  Good PSM | Beliefs about consequences (Nutrition concern, Perceived susceptibility to cancer, Benefits of FV intake), Beliefs about capabilities (Barriers to FV consumption) | **0.16**  Nutrition concern (*), Susceptibility of cancer (*), Barriers to FV consumption (*), Beta N/A |
| Glanz et al. 1998 [44] | US male workers at increased risk for colorectal cancer  (n= 2764) | Multi-component framework (SCT, TTM, HBM, Social support theory) | FV servings per day( FFQ ) | To have a healthy diet | Transver.  Low corresp.  Good BM  Low PSM | Motivation and goals (Stages of change), Beliefs about consequences (Predisposing factors), Beliefs about capabilities (Enabling factors), Sociodemographic characteristics (Age, Education, Employment status, BMI) | **0.17**  N/A |
| Houston 2001 [46] | African American Women  (n= 398) | Multi-component framework (HBM, TRA, TTM, SCT) | FV servings per day (FFQ) | Low fat food/Healthy food | Transver.  Low corresp.  Good BM  Good PSM | Knowledge (Awareness), Motivation and goals (Intention, Readiness for substituting high fat food by manufactured low-fat food, Readiness for avoiding fat, Readiness for avoiding fried food, Readiness for lowering fat content of meat, Readiness for replacement of fat food by naturally low-fat food), Beliefs in consequences (Beliefs, Perceived benefits), Social influences (Normative beliefs, Motivation to comply), Beliefs about capabilities (Label reading skills, Perceived importance of meat, Perceived importance of dessert, Skills of substitution of high fat food by manufactured low-fat food, Skills of avoiding fat, Skills of avoiding fried food, Skills of lowering fat content of meat, Skills of replacement of fat food by naturally low-fat food), Sociodemographic characteristics (Age, Education, Marital status, Number of children <18, Total income), Context and life experiences (Number of co-morbid conditions) | **0.2**  Skills of replacement of fat food (0.37***), Skills of avoid fried food (0.12*), Motivation to comply (-0.09*) |
| Krebs-Smith et al. 1995 [47] | US adults  (n=2811) | Multi-component framework (HBM, SCT) | FV servings per day (33 item FV questionnaire) | To eat FV | Transver.  Good corresp.  Good BM  Low PSM | Knowledge (Know how many portions should eat, Confident on how to choose healthy food, Advices on healthy diet are confusing), Beliefs about consequences (Prevent cancer, Prevent heart disease, Help to maintain or lose weight), Social influences (Encouragements by friends and family), Beliefs about capabilities (Influence of preparation time, Influence of price, Influence of availability, Influence of taste), Taste (Like the taste of fruits, Like the taste of vegetables), Habit (Habit to eat FV since childhood), Sociodemographic characteristics (Gender, Race, Age, Poverty category, Education), Context and life experiences (Smoking status) | **0.23**  How many servings of FV one should eat (2.61***), Habit to eat FV since childhood (0.61***), Like taste of fruits (0.65***), like taste of vegetables (0.68***) |
| Kristal et al. 1995 [48] | US adult worksite-based population  (n= 16287) | Multi-component framework (Social learning theory, TTM, Diffusion of innovations theory, HBM, Social support theory) | FV servings per day (88-items FFQ) | To increase FV | Transver.  Good corresp.  Good BM  Good PSM | Beliefs about consequences (Predisposing factors), Beliefs about capabilities (Enabling factors), Sociodemographic characteristics (Age, Gender, Race, Education, Marital status, BMI) | **0.13**  N/A |
| Moser et al. 2005 [49] | African American Men  (n= 291) | Multi-component framework (TTM, SCT) | FV servings per day (46-item FV questionnaire) | To eat more FV / to eat five FV per day | Transver.  Good corresp.  Low BM  Good PSM | Motivation and goals (No intent, Some intent, Expressed intent), Beliefs in consequences (Benefits), Social influences (Social norms, Social support), Beliefs about capabilities (Self-efficacy, Access, Cost, Others interests, Tangible reward), Taste (Dislike),Sociodemographic characteristics (Age, Marital status, Education, Household income, Employment status) | **0.22**  Benefits (0.011*), Social norms (0.004*), Tangible reward (-0.035*), Others interests (-0.055*), No intent (-0.288*) |
| Payne et al. 2005 [60] | UK company employees  (n= 286) | TPB | A total FV score (8 items FV questionnaire) | To eat healthily | Longit. (1 week)  Low corresp.  Good BM  Good PSM | Motivation and goals (Intention), Beliefs about capabilities (Perceived behavioural control), Context and life experiences (Job control, Job demands) | **0.57**  Intention (.72***), PBC (.11**) |
| Povey et al. 2000 [61] | British adults  (n= 144) | TPB | Number of FV per day (63-item FFQ | To eat 5 portions of FV per day | Longit. (1 month)  Good corresp.  Good BM  Good PSM | Motivation and goals (Intention), Beliefs about capabilities (Perceived control, self-efficacy) | **0.32**  Intention (0.42***) |
| Resnicov et al. 2000 [54] | African American church members  (n= 780) | SCT | Number of FV servings per day (means of 2, 7 and 36item-FFQ) | FV intake | Transver.  Good corresp.  Good BM  Good PSM | Knowledge, Beliefs about consequences (Outcome expectations, Perceived need, FV contain vitamins), Social influences (Social support), Beliefs about capabilities (Self-efficacy, Barriers, Meat preference, Neophobia), Social role and identity (Set example for others), Habit (High fat cooking practices, Low fat cooking practices, Vegetarian meals per month), Sociodemographic characteristics (Age, Gender, Family size, Marital status, Income, Employment status, Education level), Context and life experiences (Comorbidities, Church attendance, Smoking status, Alcohol use, Exercise, Vitamin use, Drink water) | **0.41**  Self-efficacy (0.178**), Outcome expectations (0.126**), Perceived need (-0.093**), FV contain vitamins (0.092**), Low-fat cooking practices 0.068*), Vegetarian meals (0.061**), Vitamin use (0.059**), Exercise (0.032**), Church attendance (0.028**), Drink water (0.023**), Age (0.003**) |
| Steptoe et al. 2003 [62] | UK low-income adults  (n= 271) | Multi-component framework (HBM, TPB, TTM, Self-regulation theory) | Servings of FV eaten per day (2-item FV questionnaire | To eat FV | Transver.  Good corresp.  Good BM  Good PSM | Knowledge (Knowledge of the recommended intake), Beliefs in consequences (Beliefs in health benefits, Perceived benefits), Social influences (Encouragement), Beliefs in capabilities (Perceived barriers, Self-efficacy), Sociodemographic characteristics (Age, Sex), Context and life experiences (Smoking status) | **0.13**  Knowledge (0.146*), Self-efficacy (0.14*), Perceived barriers (-0.163**) |
| Treiman 1996 [51] | US low-income women  (n= 1515) | Multi-component framework (Social learning theory, Stress model, HBM, TRA, TTM) | Daily FV intake (7 item FV questionnaire) | To eat fruits and vegetable / to eat 5 or more servings of FV | Transver.  Good corresp.  Good BM  Good PSM | Knowledge, Beliefs in consequences (Attitudes), Social influences (Social support), Beliefs about capabilities (Self-efficacy, Perceived barriers), Sociodemographic characteristics (Employment, Age, School, Number of children, Ethnicity, Marital status, Education), Context and life experiences (Site type, Responsibility for shopping, planning meals and preparing food, Cohabit-children, Cohabit-parents, Cohabit-partner, Breastfeeding, Pregnancy, Food stamps) | **0.15**  Knowledge (N/A), Social support (N/A), Perceived barriers (N/A), Responsibility for shopping, planning meals and preparing food (N/A) |
| Van Duyn et al. 2001 [55] | US adults  (n= 2605) | Multi-component framework (HBM, TTM, SCT) | FV servings per day (7-item FV questionnaire) | To adopt a diet high in FV | Transver.  Good corresp.  Good BM  Good PSM | Knowledge (Awareness), Motivation and goals (Stages of change), Social influences (Interpersonal factors), Beliefs about capabilities (Intrapersonal factors), Sociodemographic characteristics (Gender, Race, Age, Education, Income, Marital status, BMI), Context and life experiences (Smoking status, Self-rated health) | **0.37**  N/A |
| Watters et al. 2007 [52] | African American Adults  (n= 658) | Multi-component framework (SCT, TTM, Social support models) | FV servings per day( 7 item FV questionnaire ) | To eat healthy food / To eat more FV | Transver.  Low corresp.  Good BM  Low PSM | Knowledge (Knowledge of the recommended FV servings, Awareness of Food Guide Pyramid), Beliefs about consequences (Belief in FV importance, Belief that diet is related to cancer), Social influences (Encouraged, Told about good foods, Prepare healthier food with you, Eat healthier food with you), Beliefs about capabilities (Self-efficacy, Influence of cost, Influence of preparation time, Influence of difficulty to order at restaurant, Cooking skills), Taste (Taste preferences for fruits, Taste preferences for vegetables), Sociodemographic characteristics (BMI, Education, Age, Gender) | **0.11**  Knowledge (*), Belief in FV importance (***), Self-efficacy (**) Beta N/A |
| Wolf et al. 2008 [53] | African American Men  (n= 490) | Multi-component framework (HBM, TTM) | FV servings per day( 3 item FV questionnaire ) | To eat more FV/ to eat five FV per day | Transver.  Good corresp.  Good BM  Good PSM | Knowledge, Motivation and goals (Stages of change), Beliefs about consequences (Perceived benefits), Beliefs about capabilities (Perceived barriers), Sociodemographic characteristics (Age, Education, Marital status, Immigrant status) | **0.063**  Stages of change (0.13**), Knowledge (0.11*), Perceived barriers (-0.19***) |

Note: Transver.: transversal; longit.: longitudinal; corresp.: correspondence between intention and behaviour; BM: behavioural measures; PSM: psychosocial measures; N/A: not available; *p < 0.05, **p < 0.01; ***p < 0.001.
