# Supplementary material for: Psychosocial determinants of fruit and vegetable intake in adult population: a systematic review
Source: Int J Behav Nutr Phys Act. 2010 Feb 2;7:12. doi: 10.1186/1479-5868-7-12 (PMC2831029; doi:10.1186/1479-5868-7-12)
Supplement: Additional file 5 — Summary of Studies Predicting Vegetable Intake. [file 1479-5868-7-12-S5.DOC]

## Additional file 5 – Summary of Studies Predicting Vegetable Intake

| **Study** | **Population**  **(Sample Size)** | **Theoretical Framework** | **Measure of Behaviour** | **Behaviour studied by psycho-social Variables** | **Design,**  **Level of Corresp.,**  **Quality of BM,**  **Quality of PSM** | **Variables Tested** | **R2**  **Significant Variables in the Final Model** |
| --- | --- | --- | --- | --- | --- | --- | --- |
| Bogers et al. 2004 [56] | Dutch women with at least one child  (n= 159) | TPB | Vegetables in grams per day (4 item vegetable questionnaire) | To eat at least 200 grams of vegetable | Transver.  Good corresp.  Good BM  Good PSM | Motivation and goals (Intention), Beliefs about consequences (Attitude), Social influences (Subjective norm), Beliefs about capabilities (Perceived behavioural control) | **0.1**  Attitude (0.22*) |
| Brug et al. 1995 [58] | Dutch adults  (n= 367) | ASE | Vegetables in grams per day (8-item FV questionnaire) | To eat boiled vegetables | Transver.  Good corresp.  Good BM  Good PSM | Motivation and goals (Intention), Beliefs about consequences (Attitudes), Social influences, Beliefs about capabilities (Self-efficacy) | **0.12**  Intention (0.18**), Self-efficacy (0.18**) |
| Cartwright 2003 [42] | US Low-income African American women  (n= 794) | SCT | Estimates of vegetable intake (7-item FV questionnaire) | FV intake | Transver.  Low corresp.  Good BM  Good PSM | Knowledge, Beliefs about consequences (Health outcome expectancies), Beliefs about capabilities (FV availability at home, FV accessibility at home, FV accessibility away from home, Barriers, Preferences, Self-efficacy for eating 5 a day, Self-efficacy for eating FVJ when eating out, Self-efficacy for substituting high-fat food by FVJ, Self-efficacy for having FVJ available and accessible, Self-efficacy for shopping FVJ, Self-efficacy for cooking and preparing FVJ ), Social role and identity (Modeling attitudes), Past behaviour (Low-fat cooking practices, High fat cooking practices, High-fat table practices), Health value (Health as a value), Taste ( Juice preferences, Fruit preferences, Vegetable preferences), Sociodemographic characteristics (Age, Education, Employment status, Marital status, Family size), Context and life experiences ( Smoking status, Presence of current illness, Family member current illness, Family activity, Family communication, Parental involvement) | **0.14**  FV accessibility away from home (0.05*), Low-fat cooking practices (0.04*), FV availability at home (0.02**) |
| Hagler et al. 2007 [45] | US overweight and moderately obese adult men  (n= 441) | Multi-component framework (TTM, SCT) | Daily serving of vegetable/1000kcal (FFQ) | Vegetable intake | Transver.  Good corresp.  Good BM  Good PSM | Beliefs about consequences (Pros, Cons), Social influences (Social support), Beliefs about capabilities (Self-efficacy), Behavioural regulation (Change strategies) | **0.13**  Self-efficacy (0.17***), Cons (0.16***), Change strategies (0.13*) |
| Krebs-Smith et al. 1995 [47] | US adults  (n= 2811) | Multi-  component framework (HBM, SCT) | Vegetable servings per day (33 item FV questionnaire) | To eat FV | Transver.  Low corresp.  Good BM  Low PSM | Knowledge (Know how many portions should eat, Confident on how to choose healthy food, Advices on healthy diet are confusing), Beliefs about consequences (Prevent cancer, Prevent heart disease, Help to maintain or lose weight), Social influences (Encouragements by friends and family), Beliefs about capabilities (Influence of preparation time, Influence of price, Influence of availability, Influence of taste), Taste (Like the taste of fruits, Like the taste of vegetables), Past behaviour (Habit to eat FV since childhood), Sociodemographic characteristics (Gender, Race, Age, Poverty category, Education), Context and life experiences (Smoking status) | **0.23**  How many servings of FV one should eat (1.45***), Like taste of vegetables (0.67***), Habit to eat FV since childhood (0.17**) |
| Moser et al. 2005 [49] | African American Men  (n= 291) | Multi-  component framework (TTM +SCT) | Vegetable servings per day (25-item FV questionnaire) | To eat more FV/ to eat five FV per day | Transver.  Low corresp.  Low BM  Good PSM | Motivation and goals (No intent, Some intent, Expressed intent), Beliefs in consequences (Benefits), Social influences (Social norms, Social support), Beliefs about capabilities (Self-efficacy, Access, Cost, Others interests, Tangible reward), Taste (Dislike), Sociodemographic characteristics (Age, Marital status, Education, Household income, Employment status) | **0.,13**  Tangible reward (-0.032*), Other interests (-0.047*) |
| Ochs 1998 [50] | US women  (n= 211) | Multi-  component framework (HBM, SCT, TRA) | Vegetable servings per day (Block FFQ) | To adopt a healthy diet | Transver.  Low corresp.  Good BM  Low PSM | Knowledge (Nutrition awareness, Dietary advice), Motivation and goals (Nutrition intent), Beliefs about consequences (Nutrition concern), Social influences (Family influences on nutrition), Beliefs about capabilities (Barriers to change nutritional intake), Sociodemographic characteristics (Age, Household size, Family income, Education, Race, Employment, Marital status), Context and life experiences (Alcohol intake, Smoking history, Illness history, Geographic location) | **0.16**  Nutrition intent (0.008*), Geographic location (0.003*), Family income (0.01*) |
| Van Duyn et al. 2001 [55] | US adults  (n= 2605) | Multi-  component framework (HBM, TTM, SCT) | Vegetable servings per day (7-item FV questionnaire) | To adopt a diet high in FV | Transver.  Low corresp.  Good BM  Good PSM | Knowledge (Awareness), Motivation and goals (Stages of change), Social influences (Interpersonal factors), Beliefs about capabilities (Intrapersonal factors), Sociodemographic characteristics (Gender, Race, Age, Education, Income, Marital status, BMI), Context and life experiences (Smoking status, Self-rated health) | **0.32**  N/A |
| Watters et al. 2007 [52] | African American adults  (n= 658) | Multi-  component framework (SCT, TTM, Social support models) | Vegetable servings per day( 7 item FV questionnaire) | To eat healthy food / To eat more FV | Transver.  Low corresp.  Good BM  Low PSM | Knowledge (Knowledge of the recommended FV servings, Awareness of Food Guide Pyramid), Beliefs about consequences (Belief in FV importance, Belief that diet is related to cancer), Social influences (Encouraged, Told about good foods, Prepare healthier food with you, Eat healthier food with you), Beliefs about capabilities (Self-efficacy, Influence of cost, Influence of preparation time, Influence of difficulty to order at restaurant, Cooking skills), Taste (Taste preferences for fruits, Taste preferences for vegetables), Sociodemographic characteristics (BMI, Education, Age, Gender) | **0.07**  Knowledge (**), Self-efficacy (*), Taste preference for vegetable (**) Beta N/A |

Note: Transver.: transversal; longit.: longitudinal; corresp.: correspondence between intention and behaviour; BM: behavioural measures; PSM: psychosocial measures; N/A: not available; *p < 0.05, **p < 0.01; ***p < 0.001.
