# Supplementary material for: Psychosocial determinants of fruit and vegetable intake in adult population: a systematic review
Source: Int J Behav Nutr Phys Act. 2010 Feb 2;7:12. doi: 10.1186/1479-5868-7-12 (PMC2831029; doi:10.1186/1479-5868-7-12)
Supplement: Additional file 6 — Summary of Studies Explaining Fruit and Vegetable Intake Intentions. [file 1479-5868-7-12-S6.DOC]

**Additional file 6 – Summary of Studies Explaining Fruit and Vegetable Intake Intentions**

| **Study** | **Population**  **(Sample Size)** | **Theoretical Framework** | **Behaviour Studied by Psycho-social Variables** | **Measure of intention (Number of Items)** | **Quality of PSM** | **Variables Tested** | **R2**  **Significant Variables in the Final Model** |
| --- | --- | --- | --- | --- | --- | --- | --- |
| **Pertaining to Fruit and Vegetable Intake…** | | | | | | | |
| Kristal et al. 1995 [48] | US adult worksite-based population  (n= 16287) | Multi-component framework (Social Learning Theory, TTM, Diffusion of Innovations Theory,  HBM, Social Support Theory) | To eat more fruits and vegetable | 1 | Good | Beliefs about consequences (Predisposing factors), Beliefs about capabilities (Enabling factors), Sociodemographic characteristics (Age, Gender, Race, Education, Marital status, BMI) | **0.21**  N/A |
| Payne et al. 2005 [60] | UK company employees  (n= 286) | TPB | To eat healthily | 1 | Good | Beliefs about consequences (Cognitive attitude, affective attitude), Social influences (Subjective norm), Beliefs about capabilities (Perceived behavioural control), Context and life experiences (Job control, Job demands) | **0.14**  Affective attitude (0.25***), PBC (0.16**) |
| Povey et al. 2000 [61] | British adults  (n=144) | TPB | To eat at least 5 portions of fruits and vegetable per day | 3 | Good | Beliefs about consequences (Attitude, Perceived need), Social influences (Subjective norm), Beliefs about capabilities (Perceived control, Self-efficacy) | **0.68**  Self-efficacy (0.58***), Perceived need (0.46***), Attitude (0.27***), Perceived control (-0.16*), Subjective norm (0.13*) |
| **Pertaining to Fruit Intake…** | | | | | | | |
| Bogers et al. 2004 [56] | Dutch women with at least one child  (n= 159) | TPB | To eat at least 2 pieces of fruit per day | 2 | Good | Beliefs about consequences (Attitude), Social influences (Subjective norm), Beliefs about capabilities (Perceived behavioural control) | **0.44**  PBC (0.35***), Attitude (0.31***), Subjective norm (0.22**) |
| Brug et al. 1995 [58] | Dutch adults  (n= 367) | TPB | To eat fruits in the adequate amount | 1 | Good | Beliefs about consequences (Attitudes), Social influences (Social influences), Beliefs about capabilities (Self-efficacy) | **0.47**  Self-efficacy (0.69**) |
| Brug et al. 2006 [57] | Dutch adults  (n= 627) | TPB/ASE | To eat at least 2 servings of fruit per day | 2 | Good | Beliefs about consequences (Attitudes, Pros, Cons), Social influences (Subjective norm), Beliefs about capabilities (Perceived behavioural control, Situation-specific self-efficacy, Action self-efficacy, Coping self-efficacy), Past behaviour (Habit strength), Sociodemographic characteristics (Sex, Age, Education, Ethnicity) | **0.49**  Perceived Behavioural Control (0.27***), Pros (0.20***), Attitude (0.15***), Subjective Norm (0.14***), Coping self-efficacy (0.14***), Sex (0.12***), Habit (0.11***) |
| Cox et al. 1998 [63] | UK adults  (n= 741) | TPB | To increase consumption of fruit | N/A | Low | Beliefs about consequences (Attitudes), Social influences (Subjective norm), Beliefs about capabilities (Perceived control) | **0.35**  Subjective norm (0.37***), Attitudes (0.34***), Perceived control (0.12*) |
| **Pertaining to Vegetable Intake…** | | | | | | | |
| Bogers et al. 2004 [56] | Dutch women with at least one child  (n= 159) | TPB | To eat at least 200 grams of vegetable per day | 2 | Good | Beliefs about consequences (Attitude), Social influences (Subjective norm), Beliefs about capabilities (Perceived behavioural control) | **0.51**  PBC (0.52***), Attitude (0.19*), Subjective norm (0.15*) |
| Brug et al. 1995 [58] | Dutch adults  (n= 367) | ASE | To eat boiled vegetables in the adequate amount | 1 | Good | Beliefs about consequences (Attitudes), Social influences (Social influences), Beliefs about capabilities (Self-efficacy) | **0.13**  Self-efficacy (0.31**), Attitude (0.11*) |
| Cox et al. 1998 [63] | UK adults  (n= 741) | TPB | To increase consumption of vegetable | N/A | Low | Beliefs about consequences (Attitudes), Social influences (Subjective norm), Beliefs about capabilities (Perceived control) | **0.33**  Attitudes 0.34***), Subjective norm (0.32***), Perceived control (0.16***) |

Note: BM: behavioural measures; PSM: psychosocial measures; N/A: not available; *p < 0.05, **p < 0.01; ***p < 0.001
